# THE PHYSIOLOGIC COMPLEXITY OF PREFRONTAL OXYGENATION DYNAMICS IS ASSOCIATED WITH AGE AND COGNITIVE FUNCTION

**DOI:** 10.1093/geroni/igae098.2231

**Published:** 2024-12-31

**Authors:** Junhong Zhou, Yinglu Hong, Wanting Yu, Ike Iloputaife, Dapeng Bao, Brad Manor, Lewis Lipsitz, Azizah Jor’dan

**Affiliations:** Harvard Medical School/Hebrew SeniorLife, Boston, Massachusetts, United States; Beijing Sport University, Beijing, Beijing, China (People’s Republic); Hebrew SeniorLife, Boston, Massachusetts, United States; Hebrew SeniorLife, Roslindale, Massachusetts, United States; Beijing Sport University, Beijing, Beijing, China (People’s Republic); Harvard Medical School/Hebrew SeniorLife, Boston, Massachusetts, United States; Hebrew SeniorLife, Boston, Massachusetts, United States; University of Massachusetts Boston, Boston, Massachusetts, United States

## Abstract

The hemodynamics of prefrontal cortex (PFC) oxygenation are regulated by numerous elements over multiple temporal scales. The dynamics in the fluctuations of PFC oxygenation are thus complex. Age may alter the multiscale regulation of PFC oxygenation, leading to diminished physiologic complexity of this important regulatory process. We aimed to characterize the effects of age and cognitive demand on such complexity in younger and older adults and its relationship to cognitive performance. Twenty-four younger (aged 28±3 years) and 27 older adults (aged 78±6 years) completed this study. The oxygenation (HbO2) and deoxygenation (HHb) signals of the PFC were recorded using functional near-infrared-spectroscopy (fNIRS) during the n-back task of blank, identification-of-X (IdX), and 2-back conditions. We used multiscale entropy to quantify the HbO2 and HHb complexity of fNIRS. It was observed that older adults exhibited lower fNIRS complexity regardless of task condition (p=0.0005) and lower percent increase of complexity from blank (p=0.02) or IdX (p=0.01) to 2-back compared to younger. Both groups exhibited greater complexity during IdX and 2-back compared to blank condition (p< 0.02). Those with greater HbO2 complexity had greater 2-back accuracy (p=0.02~0.04). Older adults with greater fNIRS complexity had faster reaction times (β=-0.56~-0.6, p=0.009~0.02); while younger with lower complexity had faster reaction times (β=0.58~0.62, p=0.01~0.02). Participants with greater increase of fNIRS complexity from blank to 2-back had faster 2-back reaction time (β=-0.68~-0.49, p=0.001~0.03). The complexity of fNIRS-measured PFC oxygenation fluctuations may capture the influence of aging and task demands on the regulation of prefrontal hemodynamics involved in cognitive task execution.

